# Bedside lung ultrasound in the critically ill patient with pulmonary pathology: different diagnoses with comparable chest X-ray opacification

**DOI:** 10.1186/2036-7902-4-1

**Published:** 2012-03-06

**Authors:** Jacqueline Koeze, Maarten W Nijsten, Annemieke Oude Lansink, Joep M Droogh, Farouq Ismael

**Affiliations:** 1 Department of Critical Care, University Medical Center Groningen, University of Groningen, Hanzeplein 1, Groningen, 9700 RB, The Netherlands; 2 Department of Critical Care, University Medical Center Groningen, University of Groningen, Hanzeplein 1, Groningen, 9700 RB, The Netherlands

**Keywords:** ultrasound, lung, critical care, pleural effusion, consolidation

## Abstract

The differential diagnosis and treatment of opacifications on the chest X-ray in critically ill patients may be challenging. This holds in particular the patient that suffers from respiratory failure with hemodynamic instability. Opacification in the chest X-ray could be the result of hematothorax, pleural effusion, atelectasis, or consolidation. Physical examination of such patients may not always indicate what the cause of the opacification is and thus may not always help indicate the correct therapeutic approach. In such cases, bedside ultrasound may be very helpful. We present two cases with similar chest X-ray opacifications but different diagnoses established with the help of a bedside lung ultrasound. There is documented accuracy of ultrasound in differentiating pleural effusions from consolidation. Ultrasound is safe and may be an alternative for computed tomography scan in a hemodynamically or respiratory unstable intensive care patient.

## Introduction

The chest X-ray with opacification of a partial or whole lung field in critically ill patients is not uncommon. It may introduce a challenge in the differential diagnosis and may delay treatment of acute pulmonary pathology in acute respiratory failure patients. Usually, further evaluation must be performed, for example with a computed tomography (CT) scan, which can also be used to guide therapeutic interventions. A considerable number of critically ill patients are unstable to such an extent that transport to a CT scanner poses additional risks. Acting on the wrong diagnosis poses another risk. Trying to drain a pleural effusion in the absence of fluid induces the risk of pneumothorax or the risk of loss of positive end-expiratory pressure during and after pulmonary suction which leads to shearing alveolar injury and hypoxemia. An alternative reliable tool in the differential diagnosis of pulmonary pathology may be bedside lung ultrasonography.

## Case presentation

### Case 1

**Figure 1:**
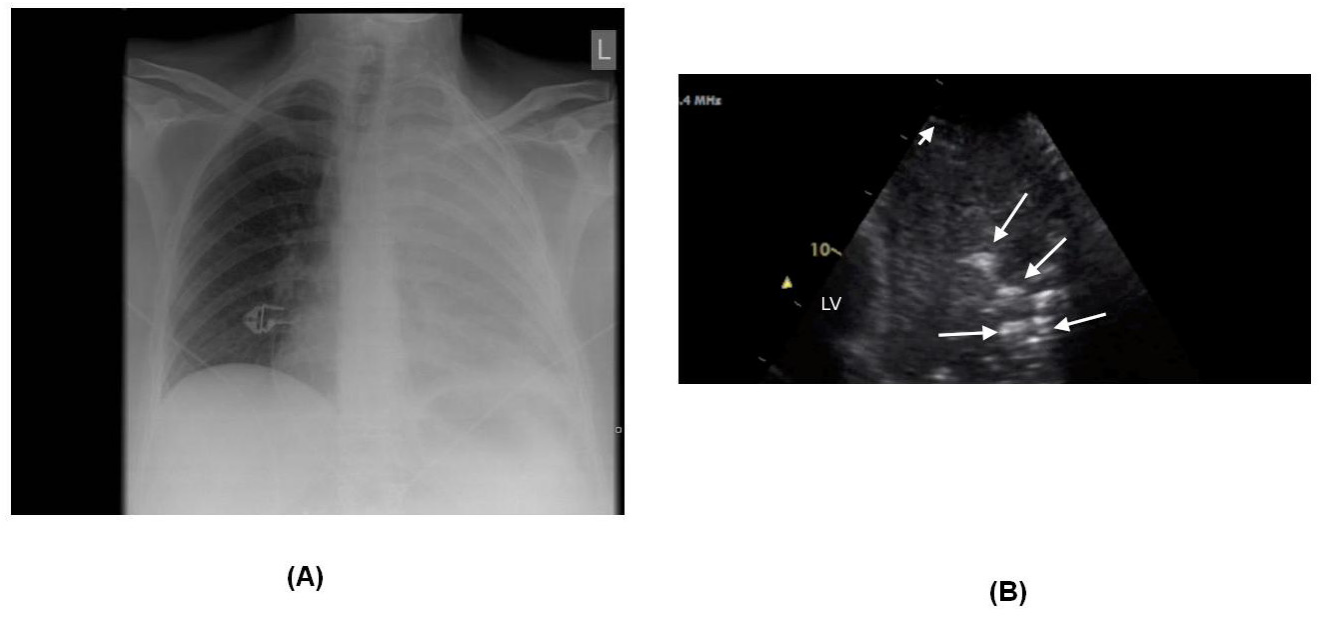
**Chest X-ray and ultrasound of patient 1**. (**A**) Chest X-ray showing complete opacification of the left lung. (**B**) Chest ultrasound showing pneumonia characterized by an irregular hypoechogenic area with air bronchograms and many hyperechogenic areas (long white arrows). The pleural line was hypoechogenic (short white arrow) as is frequently observed.

A 45-year-old woman was presented at the emergency department because of respiratory insufficiency. Her past history revealed allergic asthma with recurrent bronchitis. Five days before presentation, she developed a fever and diarrhea. The day before admission, she was seen by her general practitioner who prescribed Augmentin for a respiratory tract infection. Her chest X-ray showed complete opacification of the left lung (Figure 1A) with a differential diagnosis of pleural effusion, pneumonia, or complete left lung atelectasis. Because of progressive respiratory failure, she had to be intubated. Bedside ultrasound with a 5-MHz probe (GE FPA 2B 5 Mhz microconvex ultrasound probe, GE Healthcare Diagnostic Imaging, Hoevelaken, The Netherlands) parallel to the ribs in the anterior lower and upper thorax was conducted (Figure 1B). This revealed no pleural effusion but an irregular hypoechogenic area with air bronchograms and many hyperechogenic spots. The CT scan performed afterwards showed a practically complete consolidated left lung compatible with pneumonia.

### Case 2

**Figure 2:**
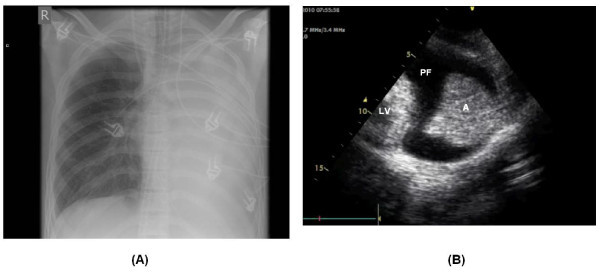
**Chest X-ray and ultrasound of patient 2**. (**A**) Chest X-ray showing total opacification of the left lung. (**B**) Lung ultrasound with atelectasis of the left lung (A) and hypoechogenic area of pleural effusion (PF).

A 16-year-old man was transferred from another hospital in cardiogenic shock which resulted from idiopathic dilated cardiomyopathy. He was supported with venous-arterial extracorporeal life support (ECLS). He required therapeutic anticoagulation because of the ECLS. There were intermittent problems with oxygenation that recovered spontaneously. After a few days, he continued to have low saturation with diminished breath sounds at the left side of the chest. A chest X-ray (Figure 2A) showed opacification of the whole left lung with the differential diagnosis of pleural effusion or total atelectasis of the left lung. Bedside ultrasound with a 5-MHz probe (GE FPA 2B 5 Mhz microconvex ultrasound probe) parallel to the ribs of the anterior lower and upper thorax revealed total left lung collapse and a surrounding hypoechogenic area suggestive of pleural effusion (Figure 2B). The pleural line was not visible in the collapsed lung. This patient was treated with bronchial suction by bronchoscopy and nursing in the right-sided position. A rigid sputum plug was removed from the left main bronchus. After this, the chest X-ray appeared nearly normal, indicating that the therapy had been effective.

## Discussion

**Figure 3:**
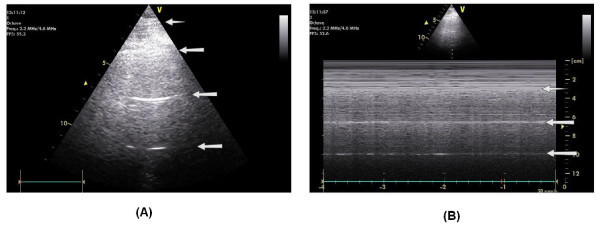
**Normal lung ultrasound**. (**A**) The pleural line (arrow): the A lines or horizontal lines arising from the pleural line are separated by regular intervals that are equal to the distance between the skin and the pleural line. (**B**) M mode shows the pleural line. Under the pleural line is the seashore sign (sandy pattern) due to the lung dynamics and pleural sliding. The horizontal lines are A lines, separated by regular interval (arrows).

The gold standard in challenging differential diagnoses of pulmonary pathology in chest X-rays is the CT scan. To reduce undue risk to the patients, like unintended extubation and dislocation of central venous catheters during transport, a noninvasive bedside tool would be preferable. The accuracy of ultrasound in diagnosing pulmonary pathology such as pleural effusion, consolidation, or pneumothorax has already been demonstrated [1,2]. In the normal lungs, ultrasound can usually identify the pleural line. The horizontal lines under the pleural line are separated by regular intervals that are equal to the distance between the skin and the pleural line. These lines are artifact lines and reflect the presence of elements with high acoustic impedance gradient (air and pleural tissue in this case), and they are called A lines (Figure 3). In a comparative study by Lichtenstein et al., it has been shown that the accuracy of ultrasound in patients with ARDS was 93% for pleural effusion, 97% for consolidation, and 95% for alveolar interstitial syndrome compared with 47%, 75%, and 72%, respectively, for chest X-rays [2]. In children, ultrasound was demonstrated to be of equal clinical value compared to CT scanning in detecting parapneumonic effusions [3]. In 2008, an algorithm (the so-called BLUE protocol) for lung ultrasound was published which reached an immediate diagnosis in acute respiratory failure of > 90% [4]. We would like to promote the use of bedside ultrasonography in the emergency department and critical care departments as a reliable, low-cost, and radiation-free tool to differentiate the main potential pulmonary diagnoses. The physical properties of the ultrasound give a good access to the pleural space pathology, such as air or fluid in the lung, or a consolidated adhesive lung. This tool has seen a significant progression in the field of critical care over the last 10 years, particularly in the case of central venous line insertion, echocardiography, and ultrasound of the lung.

## Conclusion

Lung ultrasound is a safe and reliable tool in differentiating pulmonal pathology in unstable critical care patients.

## Consent

Written informed consent was obtained from the patient for publication of this case report and any accompanying images. A copy of the written consent is available for review by the Editor-in-Chief of this journal.

## Competing interests

The authors declare that they have no competing interests.

## Authors' contributions

JK wrote the manuscript. MWN and FI edited early versions of the manuscript. AOL commented on the final version of the manuscript, and JD adapted and revised the figures. All authors read and approved the final manuscript.
